# [(*Z*)-*O*-Isopropyl *N*-(4-chloro­phen­yl)thio­carbamato-κ*S*](tricyclo­hexyl­phosphine-κ*P*)gold(I)

**DOI:** 10.1107/S1600536810009736

**Published:** 2010-03-20

**Authors:** Primjira P. Tadbuppa, Edward R. T. Tiekink

**Affiliations:** a Department of Chemistry, National University of Singapore, Singapore 117543; b Department of Chemistry, University of Malaya, 50603 Kuala Lumpur, Malaysia

## Abstract

The Au atom in the title compound, [Au(C_10_H_11_ClNOS)(C_18_H_33_P)], is coordinated within an *S*,*P*-donor set that defines a slightly distorted linear geometry [S—Au—P = 172.45 (5)°], with the distortion due in part to a close intra­molecular Au⋯O contact [3.134 (3) Å].

## Related literature

For the structural systematics and luminescence properties of phosphinegold(I) carbonimidothio­ates, see: Ho *et al.* (2006 ▶); Ho & Tiekink (2007 ▶); Kuan *et al.* (2008 ▶). For the synthesis, see Hall *et al.* (1993 ▶).
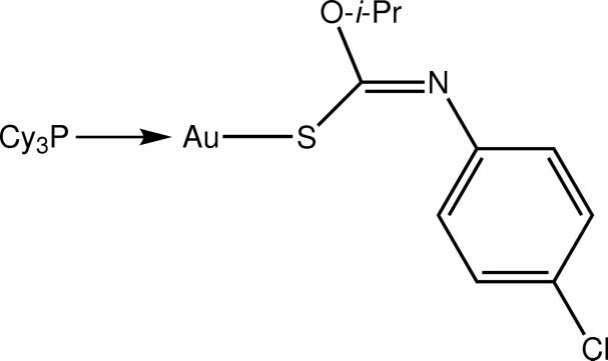


## Experimental

### 

#### Crystal data

[Au(C_10_H_11_ClNOS)(C_18_H_33_P)]
*M* *_r_* = 706.09
Monoclinic, 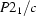

*a* = 9.4547 (15) Å
*b* = 26.137 (4) Å
*c* = 12.326 (2) Å
β = 100.162 (3)°
*V* = 2998.0 (8) Å^3^
*Z* = 4
Mo *K*α radiation
μ = 5.14 mm^−1^
*T* = 223 K
0.32 × 0.07 × 0.07 mm

#### Data collection

Bruker SMART CCD diffractometer
Absorption correction: multi-scan (*SADABS*; Bruker, 2000 ▶) *T*_min_ = 0.466, *T*_max_ = 1.000
20961 measured reflections
6886 independent reflections
5416 reflections with *I* > 2σ(*I*)
*R*_int_ = 0.037

#### Refinement

*R*[*F* ^2^ > 2σ(*F* ^2^)] = 0.039
*wR*(*F* ^2^) = 0.087
*S* = 1.06
6886 reflections
307 parameters
H-atom parameters constrained
Δρ_max_ = 3.49 e Å^−3^
Δρ_min_ = −1.06 e Å^−3^

### 

Data collection: *SMART* (Bruker, 2000 ▶); cell refinement: *SAINT* (Bruker, 2000 ▶); data reduction: *SAINT*; program(s) used to solve structure: *PATTY* in *DIRDIF92* (Beurskens *et al.*, 1992 ▶); program(s) used to refine structure: *SHELXL97* (Sheldrick, 2008 ▶); molecular graphics: *ORTEP-3* (Farrugia, 1997 ▶) and *DIAMOND* (Brandenburg, 2006 ▶); software used to prepare material for publication: *publCIF* (Westrip, 2010 ▶).

## Supplementary Material

Crystal structure: contains datablocks global, I. DOI: 10.1107/S1600536810009736/hg2659sup1.cif

Structure factors: contains datablocks I. DOI: 10.1107/S1600536810009736/hg2659Isup2.hkl

Additional supplementary materials:  crystallographic information; 3D view; checkCIF report

## Figures and Tables

**Table 1:** Selected bond lengths (Å)

Au—P1	2.2646 (12)
Au—S1	2.3027 (13)

**Fig. 1.**
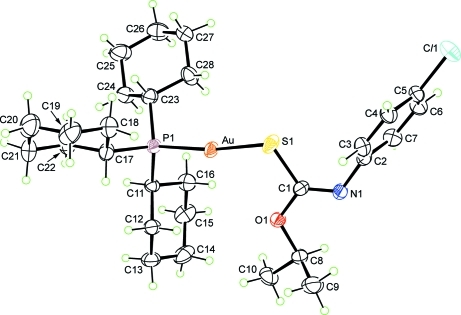
Molecular structure of (I) showing atom-labelling scheme and displacement ellipsoids at the 35% probability level.

## supplementary crystallographic information

### Comment

Crystal engineering and luminescence imperatives motivated systematic studies of R_3_PAu[SC(OR')═NR''], for R, R' and R'' = alkyl and aryl, derivatives (Ho *et al.* 2006; Ho & Tiekink, 2007; Kuan *et al.*, 2008). The synthesis and characterisation of the title compound, (I), was investigated in this context.

The gold atom in (I) exists within an *SP* donor set defined by the phosphine-*P* and thiolate-*S* atoms, Table 1 and Fig. 1. The carbonimidothioate ligand is functioning as a thiolate as seen by the magnitudes of the C1—S1 [1.750 (5) Å] and C1═N1 [1.257 (6) Å] bond distances. The coordination geometry is distorted from the ideal linear [S—Au—P = 172.45 (5) °] owing to the close approach of the O1 atom [3.134 (3) Å]. No specific intermolecular interactions are noted in the crystal packing.

### Experimental

Compound (I) was prepared following the standard literature procedure from the reaction of Cy_3_PAuCl and (*i*-Pr)OC(═S)N(H)(C_6_H_4_Cl-4) in the presence of NaOH (Hall *et al.*, 1993). Crystals were obtained by the slow evaporation of a CH_2_Cl_2_/hexane (3/1) solution held at room temperature.

### Refinement

The H atoms were geometrically placed (C—H = 0.94-0.99 Å) and refined as riding with *U*_iso_(H) = 1.2-1.5*U*_eq_(C). The maximum and minimum residual electron density peaks of 3.49 and 1.06 e Å^-3^, respectively, were located 0.85 Å and 0.70 Å from the Au atom.

### Crystal data

**Table:**

[Au(C_10_H_11_ClNOS)(C_18_H_33_P)]	*F*(000) = 1416
*M**_r_* = 706.09	*D*_x_ = 1.564 Mg m^−^^3^
Monoclinic, *P*2_1_/*c*	Mo *K*α radiation, λ = 0.71069 Å
Hall symbol: -P 2ybc	Cell parameters from 879 reflections
*a* = 9.4547 (15) Å	θ = 2.6–27.4°
*b* = 26.137 (4) Å	µ = 5.14 mm^−^^1^
*c* = 12.326 (2) Å	*T* = 223 K
β = 100.162 (3)°	Prism, colourless
*V* = 2998.0 (8) Å^3^	0.32 × 0.07 × 0.07 mm
*Z* = 4	

### Data collection

**Table:**

Bruker SMART CCD diffractometer	6886 independent reflections
Radiation source: fine-focus sealed tube	5416 reflections with *I* > 2σ(*I*)
graphite	*R*_int_ = 0.037
ω scans	θ_max_ = 27.5°, θ_min_ = 1.9°
Absorption correction: multi-scan (*SADABS*; Bruker, 2000)	*h* = −12→9
*T*_min_ = 0.466, *T*_max_ = 1.000	*k* = −33→33
20961 measured reflections	*l* = −15→15

### Refinement

**Table:**

Refinement on *F*^2^	Primary atom site location: structure-invariant direct methods
Least-squares matrix: full	Secondary atom site location: difference Fourier map
*R*[*F*^2^ > 2σ(*F*^2^)] = 0.039	Hydrogen site location: inferred from neighbouring sites
*wR*(*F*^2^) = 0.087	H-atom parameters constrained
*S* = 1.06	*w* = 1/[σ^2^(*F*_o_^2^) + (0.0354*P*)^2^ + 3.2652*P*] where *P* = (*F*_o_^2^ + 2*F*_c_^2^)/3
6886 reflections	(Δ/σ)_max_ = 0.001
307 parameters	Δρ_max_ = 3.49 e Å^−^^3^
0 restraints	Δρ_min_ = −1.06 e Å^−^^3^

### Special details

**Table:**

Geometry. All s.u.'s (except the s.u. in the dihedral angle between two l.s. planes) are estimated using the full covariance matrix. The cell s.u.'s are taken into account individually in the estimation of s.u.'s in distances, angles and torsion angles; correlations between s.u.'s in cell parameters are only used when they are defined by crystal symmetry. An approximate (isotropic) treatment of cell s.u.'s is used for estimating s.u.'s involving l.s. planes.
Refinement. Refinement of *F*^2^ against ALL reflections. The weighted *R*-factor wR and goodness of fit *S* are based on *F*^2^, conventional *R*-factors *R* are based on *F*, with *F* set to zero for negative *F*^2^. The threshold expression of *F*^2^ > 2σ(*F*^2^) is used only for calculating *R*-factors(gt) etc. and is not relevant to the choice of reflections for refinement. *R*-factors based on *F*^2^ are statistically about twice as large as those based on *F*, and *R*- factors based on ALL data will be even larger.

### Fractional atomic coordinates and isotropic or equivalent isotropic displacement parameters (Å^2^)

**Table:**

	*x*	*y*	*z*	*U*_iso_*/*U*_eq_	
Au	0.11172 (2)	0.032224 (6)	0.326336 (15)	0.03900 (7)	
Cl1	−0.41157 (16)	0.29686 (6)	0.41611 (13)	0.0629 (4)	
S1	0.04148 (19)	0.11519 (5)	0.35057 (11)	0.0547 (4)	
P1	0.16119 (15)	−0.05231 (5)	0.31795 (10)	0.0361 (3)	
O1	0.1788 (4)	0.12683 (12)	0.1876 (3)	0.0391 (8)	
N1	0.0218 (5)	0.19166 (14)	0.2031 (3)	0.0398 (9)	
C1	0.0788 (5)	0.14992 (17)	0.2375 (4)	0.0360 (10)	
C2	−0.0798 (5)	0.21535 (17)	0.2577 (4)	0.0365 (11)	
C3	−0.0419 (6)	0.23739 (19)	0.3612 (4)	0.0445 (12)	
H3	0.0537	0.2351	0.3982	0.053*	
C4	−0.1416 (6)	0.26265 (19)	0.4109 (4)	0.0431 (12)	
H4	−0.1149	0.2771	0.4813	0.052*	
C5	−0.2805 (5)	0.26620 (17)	0.3554 (4)	0.0396 (11)	
C6	−0.3205 (6)	0.2456 (2)	0.2522 (4)	0.0478 (13)	
H6	−0.4157	0.2487	0.2147	0.057*	
C7	−0.2198 (6)	0.2204 (2)	0.2044 (4)	0.0468 (12)	
H7	−0.2470	0.2063	0.1336	0.056*	
C8	0.2135 (6)	0.1506 (2)	0.0885 (4)	0.0443 (12)	
H8	0.1240	0.1610	0.0390	0.053*	
C9	0.3096 (7)	0.1964 (2)	0.1161 (6)	0.0720 (19)	
H9A	0.2581	0.2229	0.1483	0.108*	
H9B	0.3382	0.2093	0.0494	0.108*	
H9C	0.3943	0.1865	0.1683	0.108*	
C10	0.2859 (7)	0.1079 (2)	0.0358 (5)	0.0570 (15)	
H10A	0.2191	0.0798	0.0177	0.085*	
H10B	0.3696	0.0962	0.0869	0.085*	
H10C	0.3153	0.1205	−0.0309	0.085*	
C11	0.2157 (5)	−0.07089 (17)	0.1873 (4)	0.0357 (10)	
H11	0.2373	−0.1080	0.1898	0.043*	
C12	0.3503 (6)	−0.0418 (2)	0.1728 (4)	0.0467 (13)	
H12A	0.4278	−0.0496	0.2346	0.056*	
H12B	0.3313	−0.0049	0.1739	0.056*	
C13	0.3983 (7)	−0.0558 (2)	0.0650 (5)	0.0594 (16)	
H13A	0.4813	−0.0347	0.0562	0.071*	
H13B	0.4286	−0.0917	0.0678	0.071*	
C14	0.2799 (8)	−0.0479 (2)	−0.0335 (5)	0.0689 (19)	
H14A	0.3116	−0.0610	−0.0998	0.083*	
H14B	0.2610	−0.0112	−0.0438	0.083*	
C15	0.1423 (7)	−0.0750 (2)	−0.0190 (5)	0.0618 (16)	
H15A	0.0655	−0.0660	−0.0804	0.074*	
H15B	0.1569	−0.1121	−0.0206	0.074*	
C16	0.0970 (6)	−0.0603 (2)	0.0886 (4)	0.0496 (13)	
H16A	0.0108	−0.0797	0.0970	0.059*	
H16B	0.0725	−0.0239	0.0870	0.059*	
C17	0.3117 (6)	−0.07052 (19)	0.4301 (4)	0.0465 (12)	
H17	0.3989	−0.0554	0.4090	0.056*	
C18	0.2971 (7)	−0.0452 (2)	0.5377 (5)	0.0554 (14)	
H18A	0.2907	−0.0081	0.5271	0.066*	
H18B	0.2080	−0.0568	0.5602	0.066*	
C19	0.4528 (8)	−0.1131 (3)	0.6415 (7)	0.083 (2)	
H19A	0.5414	−0.1180	0.6953	0.100*	
H19B	0.3744	−0.1298	0.6706	0.100*	
C20	0.4219 (8)	−0.0573 (3)	0.6277 (5)	0.0724 (19)	
H20A	0.5077	−0.0400	0.6114	0.087*	
H20B	0.4020	−0.0435	0.6974	0.087*	
C21	0.4688 (8)	−0.1377 (3)	0.5361 (6)	0.079 (2)	
H21A	0.4788	−0.1747	0.5475	0.095*	
H21B	0.5573	−0.1252	0.5141	0.095*	
C22	0.3425 (6)	−0.1274 (2)	0.4425 (5)	0.0572 (15)	
H22A	0.3649	−0.1410	0.3733	0.069*	
H22B	0.2569	−0.1452	0.4579	0.069*	
C23	0.0073 (6)	−0.09140 (18)	0.3403 (5)	0.0458 (12)	
H23	0.0199	−0.0958	0.4212	0.055*	
C24	−0.0029 (6)	−0.14566 (19)	0.2938 (5)	0.0493 (13)	
H24A	−0.0203	−0.1442	0.2131	0.059*	
H24B	0.0883	−0.1635	0.3182	0.059*	
C25	−0.1236 (7)	−0.1750 (2)	0.3320 (6)	0.0714 (19)	
H25A	−0.1305	−0.2090	0.2980	0.086*	
H25B	−0.0997	−0.1797	0.4120	0.086*	
C26	−0.2653 (7)	−0.1494 (2)	0.3048 (6)	0.0650 (17)	
H26A	−0.3351	−0.1680	0.3401	0.078*	
H26B	−0.2985	−0.1506	0.2249	0.078*	
C27	−0.2580 (6)	−0.0941 (2)	0.3430 (5)	0.0571 (15)	
H27A	−0.3490	−0.0772	0.3129	0.068*	
H27B	−0.2470	−0.0933	0.4236	0.068*	
C28	−0.1359 (6)	−0.0642 (2)	0.3087 (5)	0.0548 (14)	
H28A	−0.1299	−0.0304	0.3437	0.066*	
H28B	−0.1561	−0.0591	0.2287	0.066*	

### Atomic displacement parameters (Å^2^)

**Table:**

	*U*^11^	*U*^22^	*U*^33^	*U*^12^	*U*^13^	*U*^23^
Au	0.05318 (13)	0.02822 (10)	0.03867 (11)	0.00624 (9)	0.01655 (8)	0.00351 (8)
Cl1	0.0515 (9)	0.0689 (9)	0.0713 (10)	0.0156 (7)	0.0188 (7)	−0.0129 (8)
S1	0.0946 (12)	0.0329 (6)	0.0458 (7)	0.0167 (7)	0.0377 (8)	0.0081 (5)
P1	0.0430 (7)	0.0294 (6)	0.0383 (7)	0.0035 (5)	0.0137 (6)	0.0014 (5)
O1	0.046 (2)	0.0363 (17)	0.0385 (18)	0.0048 (15)	0.0171 (16)	0.0017 (14)
N1	0.048 (3)	0.036 (2)	0.039 (2)	0.0080 (18)	0.0156 (19)	0.0063 (17)
C1	0.044 (3)	0.032 (2)	0.034 (2)	−0.003 (2)	0.012 (2)	0.0011 (19)
C2	0.045 (3)	0.030 (2)	0.038 (3)	0.003 (2)	0.017 (2)	0.0041 (19)
C3	0.040 (3)	0.046 (3)	0.046 (3)	0.006 (2)	0.002 (2)	0.001 (2)
C4	0.046 (3)	0.043 (3)	0.040 (3)	0.005 (2)	0.008 (2)	−0.005 (2)
C5	0.038 (3)	0.035 (2)	0.047 (3)	0.006 (2)	0.013 (2)	−0.001 (2)
C6	0.040 (3)	0.052 (3)	0.050 (3)	0.003 (2)	0.006 (2)	0.000 (2)
C7	0.048 (3)	0.051 (3)	0.041 (3)	−0.002 (2)	0.008 (2)	−0.006 (2)
C8	0.046 (3)	0.051 (3)	0.039 (3)	0.008 (2)	0.017 (2)	0.009 (2)
C9	0.069 (4)	0.053 (4)	0.105 (6)	−0.006 (3)	0.044 (4)	0.005 (4)
C10	0.058 (4)	0.068 (4)	0.049 (3)	0.015 (3)	0.022 (3)	−0.003 (3)
C11	0.038 (3)	0.031 (2)	0.040 (3)	0.000 (2)	0.015 (2)	−0.0033 (19)
C12	0.044 (3)	0.050 (3)	0.047 (3)	−0.004 (2)	0.013 (3)	0.004 (2)
C13	0.065 (4)	0.052 (3)	0.072 (4)	0.002 (3)	0.040 (4)	0.005 (3)
C14	0.108 (6)	0.057 (3)	0.052 (4)	0.001 (4)	0.043 (4)	0.003 (3)
C15	0.085 (5)	0.062 (4)	0.039 (3)	−0.014 (3)	0.011 (3)	−0.009 (3)
C16	0.045 (3)	0.063 (3)	0.039 (3)	−0.005 (3)	0.001 (2)	−0.004 (2)
C17	0.052 (3)	0.042 (3)	0.045 (3)	0.006 (2)	0.006 (2)	0.007 (2)
C18	0.059 (4)	0.052 (3)	0.052 (3)	0.003 (3)	0.002 (3)	−0.003 (3)
C19	0.067 (5)	0.096 (6)	0.079 (5)	−0.004 (4)	−0.009 (4)	0.029 (4)
C20	0.079 (5)	0.088 (5)	0.044 (3)	−0.014 (4)	−0.007 (3)	0.010 (3)
C21	0.073 (5)	0.076 (5)	0.087 (5)	0.027 (4)	0.011 (4)	0.035 (4)
C22	0.053 (4)	0.040 (3)	0.076 (4)	0.004 (3)	0.006 (3)	0.002 (3)
C23	0.050 (3)	0.036 (2)	0.055 (3)	−0.001 (2)	0.018 (3)	0.000 (2)
C24	0.049 (3)	0.039 (3)	0.063 (4)	−0.005 (2)	0.018 (3)	−0.009 (2)
C25	0.075 (5)	0.046 (3)	0.103 (6)	−0.011 (3)	0.042 (4)	−0.004 (3)
C26	0.051 (4)	0.076 (4)	0.068 (4)	−0.020 (3)	0.010 (3)	−0.010 (3)
C27	0.047 (3)	0.059 (3)	0.070 (4)	0.010 (3)	0.024 (3)	0.009 (3)
C28	0.037 (3)	0.049 (3)	0.077 (4)	0.000 (2)	0.005 (3)	−0.004 (3)

### Geometric parameters (Å, °)

**Table:**

Au—P1	2.2646 (12)	C14—H14B	0.9800
Au—S1	2.3027 (13)	C15—C16	1.514 (7)
Cl1—C5	1.751 (5)	C15—H15A	0.9800
S1—C1	1.750 (5)	C15—H15B	0.9800
P1—C23	1.838 (5)	C16—H16A	0.9800
P1—C11	1.840 (4)	C16—H16B	0.9800
P1—C17	1.862 (5)	C17—C18	1.511 (8)
O1—C1	1.356 (5)	C17—C22	1.518 (7)
O1—C8	1.459 (5)	C17—H17	0.9900
N1—C1	1.257 (6)	C18—C20	1.503 (8)
N1—C2	1.410 (6)	C18—H18A	0.9800
C2—C7	1.376 (7)	C18—H18B	0.9800
C2—C3	1.388 (7)	C19—C21	1.481 (10)
C3—C4	1.380 (7)	C19—C20	1.492 (10)
C3—H3	0.9400	C19—H19A	0.9800
C4—C5	1.373 (7)	C19—H19B	0.9800
C4—H4	0.9400	C20—H20A	0.9800
C5—C6	1.371 (7)	C20—H20B	0.9800
C6—C7	1.374 (7)	C21—C22	1.531 (9)
C6—H6	0.9400	C21—H21A	0.9800
C7—H7	0.9400	C21—H21B	0.9800
C8—C9	1.505 (8)	C22—H22A	0.9800
C8—C10	1.514 (7)	C22—H22B	0.9800
C8—H8	0.9900	C23—C28	1.518 (7)
C9—H9A	0.9700	C23—C24	1.526 (7)
C9—H9B	0.9700	C23—H23	0.9900
C9—H9C	0.9700	C24—C25	1.516 (7)
C10—H10A	0.9700	C24—H24A	0.9800
C10—H10B	0.9700	C24—H24B	0.9800
C10—H10C	0.9700	C25—C26	1.481 (9)
C11—C12	1.520 (7)	C25—H25A	0.9800
C11—C16	1.528 (7)	C25—H25B	0.9800
C11—H11	0.9900	C26—C27	1.519 (8)
C12—C13	1.524 (7)	C26—H26A	0.9800
C12—H12A	0.9800	C26—H26B	0.9800
C12—H12B	0.9800	C27—C28	1.514 (7)
C13—C14	1.513 (9)	C27—H27A	0.9800
C13—H13A	0.9800	C27—H27B	0.9800
C13—H13B	0.9800	C28—H28A	0.9800
C14—C15	1.519 (9)	C28—H28B	0.9800
C14—H14A	0.9800		
			
P1—Au—S1	172.45 (5)	C15—C16—C11	111.7 (5)
C1—S1—Au	106.36 (16)	C15—C16—H16A	109.3
C23—P1—C11	109.6 (2)	C11—C16—H16A	109.3
C23—P1—C17	105.6 (2)	C15—C16—H16B	109.3
C11—P1—C17	106.7 (2)	C11—C16—H16B	109.3
C23—P1—Au	111.10 (16)	H16A—C16—H16B	107.9
C11—P1—Au	112.92 (15)	C18—C17—C22	112.7 (5)
C17—P1—Au	110.63 (17)	C18—C17—P1	111.3 (4)
C1—O1—C8	117.9 (4)	C22—C17—P1	115.7 (4)
C1—N1—C2	120.5 (4)	C18—C17—H17	105.4
N1—C1—O1	121.7 (4)	C22—C17—H17	105.4
N1—C1—S1	125.7 (4)	P1—C17—H17	105.4
O1—C1—S1	112.6 (3)	C20—C18—C17	112.0 (5)
C7—C2—C3	118.0 (4)	C20—C18—H18A	109.2
C7—C2—N1	119.6 (4)	C17—C18—H18A	109.2
C3—C2—N1	122.2 (5)	C20—C18—H18B	109.2
C4—C3—C2	121.4 (5)	C17—C18—H18B	109.2
C4—C3—H3	119.3	H18A—C18—H18B	107.9
C2—C3—H3	119.3	C21—C19—C20	111.8 (6)
C5—C4—C3	118.7 (5)	C21—C19—H19A	109.3
C5—C4—H4	120.7	C20—C19—H19A	109.3
C3—C4—H4	120.7	C21—C19—H19B	109.3
C6—C5—C4	121.3 (5)	C20—C19—H19B	109.3
C6—C5—Cl1	118.4 (4)	H19A—C19—H19B	107.9
C4—C5—Cl1	120.4 (4)	C19—C20—C18	113.7 (6)
C5—C6—C7	119.2 (5)	C19—C20—H20A	108.8
C5—C6—H6	120.4	C18—C20—H20A	108.8
C7—C6—H6	120.4	C19—C20—H20B	108.8
C6—C7—C2	121.5 (5)	C18—C20—H20B	108.8
C6—C7—H7	119.3	H20A—C20—H20B	107.7
C2—C7—H7	119.3	C19—C21—C22	113.5 (6)
O1—C8—C9	111.5 (5)	C19—C21—H21A	108.9
O1—C8—C10	103.3 (4)	C22—C21—H21A	108.9
C9—C8—C10	112.5 (5)	C19—C21—H21B	108.9
O1—C8—H8	109.8	C22—C21—H21B	108.9
C9—C8—H8	109.8	H21A—C21—H21B	107.7
C10—C8—H8	109.8	C17—C22—C21	111.0 (5)
C8—C9—H9A	109.5	C17—C22—H22A	109.4
C8—C9—H9B	109.5	C21—C22—H22A	109.4
H9A—C9—H9B	109.5	C17—C22—H22B	109.4
C8—C9—H9C	109.5	C21—C22—H22B	109.4
H9A—C9—H9C	109.5	H22A—C22—H22B	108.0
H9B—C9—H9C	109.5	C28—C23—C24	110.1 (5)
C8—C10—H10A	109.5	C28—C23—P1	113.3 (4)
C8—C10—H10B	109.5	C24—C23—P1	117.3 (4)
H10A—C10—H10B	109.5	C28—C23—H23	104.9
C8—C10—H10C	109.5	C24—C23—H23	104.9
H10A—C10—H10C	109.5	P1—C23—H23	104.9
H10B—C10—H10C	109.5	C25—C24—C23	110.8 (4)
C12—C11—C16	109.2 (4)	C25—C24—H24A	109.5
C12—C11—P1	109.9 (3)	C23—C24—H24A	109.5
C16—C11—P1	111.7 (3)	C25—C24—H24B	109.5
C12—C11—H11	108.7	C23—C24—H24B	109.5
C16—C11—H11	108.7	H24A—C24—H24B	108.1
P1—C11—H11	108.7	C26—C25—C24	113.7 (5)
C11—C12—C13	111.5 (4)	C26—C25—H25A	108.8
C11—C12—H12A	109.3	C24—C25—H25A	108.8
C13—C12—H12A	109.3	C26—C25—H25B	108.8
C11—C12—H12B	109.3	C24—C25—H25B	108.8
C13—C12—H12B	109.3	H25A—C25—H25B	107.7
H12A—C12—H12B	108.0	C25—C26—C27	111.7 (5)
C14—C13—C12	112.0 (5)	C25—C26—H26A	109.3
C14—C13—H13A	109.2	C27—C26—H26A	109.3
C12—C13—H13A	109.2	C25—C26—H26B	109.3
C14—C13—H13B	109.2	C27—C26—H26B	109.3
C12—C13—H13B	109.2	H26A—C26—H26B	107.9
H13A—C13—H13B	107.9	C28—C27—C26	113.6 (5)
C13—C14—C15	111.8 (5)	C28—C27—H27A	108.9
C13—C14—H14A	109.3	C26—C27—H27A	108.9
C15—C14—H14A	109.3	C28—C27—H27B	108.9
C13—C14—H14B	109.3	C26—C27—H27B	108.9
C15—C14—H14B	109.3	H27A—C27—H27B	107.7
H14A—C14—H14B	107.9	C27—C28—C23	112.1 (5)
C16—C15—C14	111.5 (5)	C27—C28—H28A	109.2
C16—C15—H15A	109.3	C23—C28—H28A	109.2
C14—C15—H15A	109.3	C27—C28—H28B	109.2
C16—C15—H15B	109.3	C23—C28—H28B	109.2
C14—C15—H15B	109.3	H28A—C28—H28B	107.9
H15A—C15—H15B	108.0		
			
P1—Au—S1—C1	−159.3 (4)	C12—C13—C14—C15	−52.6 (7)
S1—Au—P1—C23	29.2 (5)	C13—C14—C15—C16	53.0 (7)
S1—Au—P1—C11	152.8 (4)	C14—C15—C16—C11	−56.1 (7)
S1—Au—P1—C17	−87.8 (5)	C12—C11—C16—C15	57.6 (6)
C2—N1—C1—O1	−178.8 (4)	P1—C11—C16—C15	179.3 (4)
C2—N1—C1—S1	1.4 (7)	C23—P1—C17—C18	−78.2 (4)
C8—O1—C1—N1	−3.1 (7)	C11—P1—C17—C18	165.2 (4)
C8—O1—C1—S1	176.7 (3)	Au—P1—C17—C18	42.1 (4)
Au—S1—C1—N1	158.6 (4)	C23—P1—C17—C22	52.1 (5)
Au—S1—C1—O1	−21.1 (4)	C11—P1—C17—C22	−64.4 (5)
C1—N1—C2—C7	−114.7 (6)	Au—P1—C17—C22	172.4 (4)
C1—N1—C2—C3	70.2 (6)	C22—C17—C18—C20	51.2 (7)
C7—C2—C3—C4	1.5 (7)	P1—C17—C18—C20	−177.0 (4)
N1—C2—C3—C4	176.7 (4)	C21—C19—C20—C18	52.5 (9)
C2—C3—C4—C5	−0.7 (8)	C17—C18—C20—C19	−51.8 (8)
C3—C4—C5—C6	−0.6 (8)	C20—C19—C21—C22	−52.7 (8)
C3—C4—C5—Cl1	178.8 (4)	C18—C17—C22—C21	−50.9 (7)
C4—C5—C6—C7	0.9 (8)	P1—C17—C22—C21	179.4 (4)
Cl1—C5—C6—C7	−178.6 (4)	C19—C21—C22—C17	52.1 (8)
C5—C6—C7—C2	0.1 (8)	C11—P1—C23—C28	−99.4 (4)
C3—C2—C7—C6	−1.2 (7)	C17—P1—C23—C28	146.1 (4)
N1—C2—C7—C6	−176.5 (5)	Au—P1—C23—C28	26.1 (5)
C1—O1—C8—C9	76.9 (6)	C11—P1—C23—C24	30.7 (5)
C1—O1—C8—C10	−162.0 (4)	C17—P1—C23—C24	−83.8 (5)
C23—P1—C11—C12	−175.4 (3)	Au—P1—C23—C24	156.2 (4)
C17—P1—C11—C12	−61.6 (4)	C28—C23—C24—C25	−56.0 (7)
Au—P1—C11—C12	60.1 (4)	P1—C23—C24—C25	172.5 (4)
C23—P1—C11—C16	63.3 (4)	C23—C24—C25—C26	55.8 (8)
C17—P1—C11—C16	177.1 (4)	C24—C25—C26—C27	−51.6 (8)
Au—P1—C11—C16	−61.2 (4)	C25—C26—C27—C28	49.3 (8)
C16—C11—C12—C13	−56.7 (6)	C26—C27—C28—C23	−51.7 (7)
P1—C11—C12—C13	−179.5 (4)	C24—C23—C28—C27	54.6 (6)
C11—C12—C13—C14	55.2 (6)	P1—C23—C28—C27	−171.8 (4)
